# A Comparative Study of Melanocytic Tumours: Linking Portuguese Dogs and Cats to Human Cases

**DOI:** 10.1111/vco.70031

**Published:** 2025-12-10

**Authors:** Catarina Alves Pinto, Ana Isabel Ribeiro, João Niza‐Ribeiro, Carlos Alberto Palmeira de Sousa, Katia Pinello, Andreia Alexandra Ferreira Santos

**Affiliations:** ^1^ Department of Veterinary Clinics, ICBAS–School of Medicine and Biomedical Sciences University of Porto Porto Portugal; ^2^ Experimental Pathology and Therapeutics Research Group of the Research Center of IPO‐Porto Porto Portugal; ^3^ Departamento de Geografia Faculdade de Letras da Universidade do Porto, Centro de Estudos em Geografia e Ordenamento do Território (CEGOT) Porto Portugal; ^4^ EPIUnit ITR, Institute of Public Health of the University Porto University of Porto (ISPUP) Porto Portugal; ^5^ Laboratório Para a Investigação Integrativa e Translacional em Saúde Populacional (ITR) Porto Portugal; ^6^ Vet‐OncoNet, Population Studies Department, ICBAS–School of Medicine and Biomedical Sciences University of Porto Porto Portugal; ^7^ Clinical Pathology Service (Immunology Department) of the IPO‐Porto Porto Portugal; ^8^ I3ID and Health School of University Fernando Pessoa Porto Portugal; ^9^ Animal Science and Study Centre, Food and Agrarian Sciences and Technologies Institute (CECA‐ICETA) University of Porto Porto Portugal

**Keywords:** cat, comparative oncology, dog, epidemiology, geographic distribution, human, melanocytic tumours

## Abstract

Melanocytic tumours (MT) occur in both humans and companion animals, presenting an opportunity for comparative oncology research. Thus, this study provides a comprehensive epidemiological analysis comparing MT in Portuguese dogs, cats and humans. Data were obtained from the Portuguese National Cancer Registry (RON) (2011–2021) and Vet‐OncoNet (2020–2023), utilising standardised oncological classification systems (ICD‐O‐3.2 and Vet‐ICD‐O‐canine‐1). The results indicate that Melanoma was the most frequently diagnosed MT across all three species, while melanocytomas were common in dogs but rare in cats and humans. A higher incidence rate (IR) for MT was observed in dogs (IR = 16.1) compared to humans (IR = 8.1) and cats (IR = 6.3), and neutered dogs (10.8 years) were diagnosed at significantly older ages than intact ones (9.9 years). Shar‐Peis (RR = 14.2, *p* < 0.001) had the highest RR compared to mixed‐breed dogs, followed closely by Rhodesian Ridgebacks (RR = 12.2, *p* < 0.001) and Golden Retrievers (RR = 6.4, *p* < 0.001). Spatial analysis revealed significant clustering of MT cases in humans and dogs, with a strong geographical overlap (BLISA = 0.345, *p* < 0.001) in urban regions. This study provides the first epidemiological comparison of MT in these three species in Portugal, underscoring the sentinel role of companion animals in human oncology and the relevance of comparative oncology in translational cancer research.

## 1 Introduction

Cancer is a leading cause of death globally, particularly in developed countries, with environmental carcinogens responsible for 1.3 million new cases annually [1, 2, 3]. Advances in comparative oncology show that dogs and cats, that live with humans as companion animals, can be valuable models for human cancer, as they share the same environment and exposure to risk factors [4, 5, 6, 7, 8]. With shorter lifespans, companion animals can serve as sentinels for reducing human cancer incidence [9, 10, 11, 12, 13, 14].

In humans, melanocytic neoplasms frequently occur in the skin; however, they can also appear in several internal organs, including the central nervous system [15]. Benign melanocytic tumours in humans are generally designated by melanocytic nevi (plural of nevus), and the malignant ones by melanoma [15]. Even though melanomas can originate from nevi, most primary melanomas do not have a detectable associated precursor nevus [15].

Melanoma is presently the fastest increasing incident cancer, and several studies into its aetiology suggest multiple causes independent of the previously mentioned UV‐associated mutagenesis [12]. Although the skin is the most typical location, cutaneous melanomas represent less than 5% of skin neoplasms, but due to their aggressive behaviour and high metastatic rate, they are the cause of the majority of deaths originated from cutaneous neoplastic malignancies [12].

Melanocytic tumours (MT) are increasingly frequent in dogs, especially oral melanoma, which is the most common oral malignant neoplasm in this species [3]. In dogs, 70% of MT are malignant melanoma and account for 7% of all malignant tumours [3]. Melanocytomas, the benign form of MT, account for 30% of these tumours [4]. Dog breeds that are heavily pigmented and middle‐aged to senior dogs are the most commonly affected [3, 4]. In dogs, 62% of the melanomas are located in the oral cavity, 27% are cutaneous, 6% are digital, and 4% are subungual [4]. Ocular, nasal cavity, footpads, anal sacs, and gastrointestinal tract melanomas have also been reported [3]. Oral melanoma represents 14.4% to 45.5% of all oral tumours, being the most frequent oral neoplasm in dogs [3, 16]. Considering all canine skin tumours, cutaneous melanoma represents 0.8% to 2% of these neoplasms [16]. Ocular MTs in dogs exhibit different phenotypes: tumours located in the conjunctiva are normally malignant, while uveal, limbal, and iridal tumours are mostly benign [17, 18].

MTs are rare in cats, accounting for less than 1% of all malignancies, mostly intraocular [3, 19]. They represent 0.8% to 7% of feline cutaneous tumours and < 1% of oral neoplasms [20, 21]. These tumours occur mainly in the eye (intraocular and limbus), haired skin (especially the pinna), and oral cavity, with some reports of nasal planum melanoma [22]. Affected cats are typically 11–13 years old, although pinna melanomas occur in younger cats, with a median age of 7 [20]. No sex or breed predisposition has been reported [20].

To date, no comparative studies exist on MTs between humans, dogs and cats, nor on their geographic distribution in Portugal. Therefore, this study aims to describe and compare MTs across these species, focusing on epidemiological indicators, geographic distribution, and potential risk factors.

## 2 Materials and Methods

### 2.1 Cell Line Validation Statement

No cell lines were used in this study.

### 2.2 Study Design

This is a cross‐sectional retrospective study, comparing the geographic distribution and incidence risks of MTs in dogs, cats, and humans, within the different districts of Portugal. This study was approved by the Animal Welfare Ethics Committee of ICBAS–School of Medicine and Biomedical Sciences, University of Porto, Portugal (reference P394/2021/ORBEA). As per rules of the Portuguese Oncology Registry (RON), data from human cases were requested to the Ethics Committee of the Portuguese Institute of Oncology (IPO) in Porto with the reference number CES 039/024.

### 2.3 Data Collection

#### 2.3.1 Human Data

Human MTs cases, between 2011 and 2021, were obtained for all the Portuguese districts. The data collected included date of diagnosis, age, sex, municipality (i.e., area of residence of the person), tumour morphology (histological type), topography, tumour behaviour, classified according to the International Classification of Diseases for Oncology, 3rd edition (ICD‐O‐3.2) [23].

Data on the resident human population was obtained from the Portuguese National Institute of Statistics, based on the population estimates from 2011 to 2021 [24].

Regarding morphology, humans have several subtypes of malignant MTs (coded as /3 in ICD‐O‐3.2) apart from melanoma and amelanotic melanoma (see Table S1), so it was decided to include all the malignant tumours (except for the amelanotic melanoma) in the melanoma group, in order to compare them in an easier way with the dogs' and cats' MTs groups, as these species do not have as many MT subtypes as humans.

#### 2.3.2 Animal Data

Canine and feline MTs cases from 2020 to 2023 were obtained from the Portuguese Veterinary Cancer Registry (Vet‐OncoNet) platform [25]. The collected data included breed, sex, age, postal code of the veterinary centres where the animal was diagnosed and followed, tumour morphology, topography, behaviour and date of diagnosis, and were classified according to the Veterinary International Classification of Diseases for Oncology‐canine (Vet‐ICD‐O‐canine), 1st edition [26].

Data on the dog and cat population was obtained from the Portuguese Companion Animal Information System (SIAC), which is the official Portuguese registry of companion animals [27]. Vet‐OncoNet oversees the data management of SIAC, utilising records of living dogs and cats registered in the database from 2004 to 2023 (data not shown). The mixed‐breed dogs were used as a reference for comparison with other dog breeds, as they are the predominant population among dogs.

### 2.4 Data Analysis

After performing internal validity checks and data cleaning using Microsoft Office Excel 2013, the analysis was conducted using R, version 4.1.2.

A descriptive analysis was performed, focusing on age, sex, breed (for both dogs and cats), tumour morphology, and topography. Numerical summaries were presented as frequencies and percentages. Continuous variables were reported as means and standard deviations, while categorical variables were expressed as counts and percentages.

### 2.5 Age Analysis

Differences in mean age were assessed using Student's *T*‐test and ANOVA, with post hoc comparisons performed using the Tukey test. For comparative analysis of the ages at diagnosis for MTs in humans, dogs, and cats, a *Z*‐score was calculated. Wilcoxon rank‐sum tests were employed to assess differences in the medians of the *Z*‐scores, while an *F*‐test was used to evaluate variances. Additionally, two‐sample Kolmogorov–Smirnov tests were conducted to compare the distributions of the *Z*‐scores. The Kolmogorov–Smirnov test was chosen for *Z*‐score comparisons because it is a non‐parametric method that evaluates differences in the cumulative distribution functions of two samples. This approach is appropriate given the potential deviations from normality in *Z*‐score distributions across species.

### 2.6 Incidence Rates

For human cases, the mean annual incidence rate (IR) was calculated to estimate the frequency of new cases, adjusted for the observation period (2011–2021). The total number of cases reported over the 11‐year period was divided by the number of years to obtain the mean annual number of cases. This value was then divided by the mean estimated population between 2011 and 2021 and multiplied by 100 000 to express the incidence rate per 100 000 individuals.

For dogs and cats, the IR was calculated by dividing the total number of cases by four and then dividing by the total population registered in SIAC in 2023 and multiplied by 100 000 to express the incidence rate per 100 000 animals. IR was calculated for all MTs, benign tumours (melanocytomas for animals and nevi for humans), and malignant tumours (melanomas and amelanotic melanomas) (Table S1). The IR 95% confidence interval (95% CI) was calculated, applying an exact method based on the Poisson distribution to account for variability in the observed data.

### 2.7 Relative Risks

Relative Risks (RRs) were calculated to assess breed‐specific risks to MTs. Due to the lack of accuracy in cat breed identification, relative risks were calculated exclusively for dogs. The RRs were determined for each breed and categorised by all MTs, amelanotic melanomas, benign tumours and melanomas, using mixed‐breed dogs as the reference group, due to their genetic diversity, large population size, and suitability for assessing breed‐specific risks. Proportional differences were analysed using the Chi‐square test, with 95% CI.

### 2.8 Comparative Analysis

For comparative analysis, the age‐standardised incidence rate (ASIR) for humans and dogs was calculated by considering the number of cases and the corresponding population within each municipality and age category. The indirect standardisation method was applied, using the human and canine populations of Portugal as the reference.

### 2.9 Spatial Analysis

Spatial analysis was conducted in GeoDa version 1.20 and mapping was conducted in ArcGIS Pro 3.1.0. GeoDa was employed to analyse spatial autocorrelation and identify clusters. Global spatial autocorrelation was assessed using Moran's Index (Moran's I) with Empirical Bayes (EB) smoothing, while local clusters were detected using the Local Index of Spatial Autocorrelation (LISA) with EB. To perform these analyses, an EB rate was first calculated by smoothing the standardised incidence ratio (SIR). This was derived from the ‘event’ variable (observed cases) and the ‘base’ variable (expected cases), providing a more stable estimate of the IRs of MTs in humans and dogs. The resulting smoothed SIR was then utilised for both global and local Moran's statistics. The global Moran's *I* statistic was used to measure the overall degree of clustering. A global Moran's *I* > 0 indicates a clustered pattern (i.e., similar values are found near each other), *I* = 0 indicates a random pattern, and *I* < 0 indicates a dispersed pattern.

Then, the LISA was calculated for each municipality in the study area to identify spatial clusters of similar MTs rates. It allows us to identify whether a municipality was part of a cluster of similar values (high‐high or low‐low) or was surrounded by dissimilar values (high‐low or low‐high). High‐low outliers are municipalities with high rates surrounded by municipalities with low rates, while low‐high outliers are those with low rates surrounded by municipalities with high rates.

Additionally, we also calculated the Bivariate Moran's (BLISA) to examine the spatial relationship between the SIR of humans and dogs simultaneously. It determines whether there is a spatial association between the values of the two variables, helping to identify patterns of similarity in their geographical distribution.

For all statistical analyses, a *p* value below 0.05 was deemed indicative of statistical significance.

## 3 Results

### 3.1 Melanocytic Tumours in Humans

A total of 18 324 cases of MTs in human patients across Portugal were included in this study. When it comes to sex, women represented the majority, accounting for 54.9% of cases (10050), while men made up 45.2% (8274).

Looking at age distribution (see Table 1), the most common affected age group was 45–69 years, which comprised 44.3% of all cases. In terms of tumour location (Table 1), the skin (C44) was by far the most frequent site, accounting for 92.6% of cases. The next most common locations were the eyes and adnexa (C69), which represented 3.2% of the cases.

**TABLE 1:** Descriptive analysis of MTs in humans, registered in the Portuguese Oncology Registry (RON) database, between 2011 and 2021.

	Total		Women		Men	
	*n*	% (in column)	n	% (in line)	*n*	% (in line)
Total	18 324	100	10 050	54.9	8274	45.2
Age						
0–19	231	1.3	137	59.3	94	40.7
20–44	2920	15.9	1840	63	1080	37
45–69	8119	44.3	4409	54.3	3710	45.7
≥ 70	7021	38.3	3644	51.9	3377	48.1
Missings	33	0.2	20	60.6	13	39.4
Morphology						
Malignant melanomas^a^	17 469	95.3	9529	54.5	7940	45.5
Benign tumours—nevus^a^	830	4.5	507	61.1	323	38.9
Amelanotic melanoma	25	0.1	14	56	11	44
Topography						
Skin_(C44.‐ and C49.‐)_	16 974	92.6	9301	54.8	7673	45.2
Eyes and adnexas_(C69.‐)_	582	3.2	296	50.9	286	49.1
Unknown primary site_(C.80)_	353	1.9	171	48.4	182	51.6
Digestive organs and peritoneum_(C15.‐C.26; C48)_	116	0.6	70	60.3	46	39.7
Respiratory and thoracic organs_(C30.‐ to C39.‐)_	115	0.6	78	67.8	37	32.2
Female genitals_(C51.‐ to C58.)_	100	0.6	100	100		0
Oropharyngeal cavity_(C00.‐ to C14.‐)_	37	0.2	17	45.9	20	54.1
Connective, subcutaneous and other soft tissues_(C49.)_	13	0.1	4	30.8	9	69.2
Male genitals_(C60.‐ to C63.‐)_	11	0.1		0	11	100
Nervous system_(C47.; C70.‐C72.)_	9	0.1	4	44.4	5	55.6
Urinary organs_(C64.‐C68.)_	8	0	5	62.5	3	37.5
Lymph nodes_(C77.)_	3	0	2	66.7	1	33.3
Mammary gland_(C50.‐)_	2	0	1	50	1	50
Bone and joints_(C40.‐ and C41)_	1	0	1	100	0	0

^a^ See Table S1 to check all subtypes included in this group.

In terms of tumour types (Table 1), the most commonly diagnosed form of MT was Malignant melanoma, NOS (8720/3), making up 45.4% (8319 cases). This was followed by low cumulative sun damage melanoma (8743/3), which accounted for 27.2% (4986 cases) of the diagnoses.

MTs were notably more prevalent in women, who also showed a higher incidence of both melanomas and benign tumours. In terms of tumour location, these cases were most commonly found in the skin, digestive organs, and peritoneum, as well as in the respiratory and thoracic organs.

### 3.2 Melanocytic Tumours in Dogs and Cats

A total of 1303 cases of MTs were included in the study, with 1199 cases from dogs (92.0%) and 104 from cats (8.0%), spanning from January 2019 to December 2023.

In dogs, males were more commonly affected (see Table 2), with the most frequent age range being 8 to 11 years. In cats, females predominated (see Table 2), and, like dogs, the 8‐ to 11‐year age range was the most commonly observed for tumour development. MTs in dogs were most commonly found in the skin, followed by the oropharyngeal cavity. In terms of tumour types (see Table 2), Melanoma, NOS was the most prevalent, followed by melanocytoma. In cats, the skin was also the primary site for MTs, followed by the eyes and adnexa. Similar to dogs, Melanoma, NOS was the most frequently observed tumour, followed by melanocytoma.

**TABLE 2:** Descriptive analysis of MTs in dogs and cats, registered in the Vet‐OncoNet database, between 2019 and 2023.

	Total		Dogs						Cats					
	*n*	% (in column)	Total	% (in column)	F	% (in line)	M	% (in line)	Total	% (in column)	F	% (in line)	M	% (in line)
Total	1303	100	1199	92	533	44.4	660	55.1	104	8	56	53.8	46	44.2
Age														
0–3	33	2.5	25	2.1	14	56	8	32	8	7.7	5	62.5	2	25
4–7	228	17.5	209	17.4	89	42.6	90	43.1	19	18.3	10	52.6	7	36.8
8–11	534	41	498	41.5	203	40.8	281	56.4	36	34.6	16	44.4	20	55.6
> 12	399	30.6	366	30.5	159	43.4	195	53.3	33	31.7	21	63.6	11	33.3
Missings	109	8.4	101	8.4	39	38.6	60	59.4	8	7.7	3	37.5	3	37.5
Morphology_(Vet‐ICD‐O)_														
Melanoma, NOS_(8720/3)_	609	46.7	526	43.9	222	42.2	293	55.7	83	79.8	46	55.4	36	43.4
Melanocytoma_(8720.0/0)_	524	40.2	510	42.5	234	45.9	272	53.3	14	13.5	8	57.1	5	35.7
Amelanotic melanoma_(8730/3)_	168	12.9	161	13.4	72	44.7	89	55.3	7	6.7	2	28.6	5	71.4
Melanoacanthoma_(8726.1/0)_	2	0.2	2	0.2	0	0	2	100	0	0	0	0	0	0
Topography														
Skin_(C44.‐ and C49.‐)_	820	62.9	772	64.4	367	47.5	401	51.9	48	46.2	28	58.3	19	39.5
Oropharyngeal cavity_(C00.‐ to C14.‐)_	313	24	304	25.4	114	36.4	189	62.2	9	8.7	4	44.4	5	55.6
Eyes and adnexas_(C69.‐)_	105	8.1	60	5	32	53.3	27	45	45	43.3	23	51.1	21	46.7
Male genitals_(C60.‐ to C63.‐)_	26	2	26	2.2	0	0	26	100	0	0	0	0	0	0
Unknown primary site_(C.80)_	26	2	25	2.1	12	48	13	52	1	1	1	100	0	0
Bone and joints_(C40.‐ and C41)_	5	0.4	5	0.4	3	60	2	40	0	0	0	0	0	0
Respiratory and thoracic organs_(C30.‐ to C39.‐)_	4	0.3	3	0.3	2	66.7	1	33.3	1	1	0	0	1	100
Mammary gland_(C50.‐)_	3	0.2	3	0.3	2	50	1	25	0	0	0	0	0	0
Female genitals_(C51.‐ to C58.)_	1	0.1	1	0.1	1	100	0	0	0	0	0	0	0	0

Abbreviations: F, females; M, males.

### 3.3 Sex, Reproductive Status, and Breed in Dogs

**FIGURE 1:**
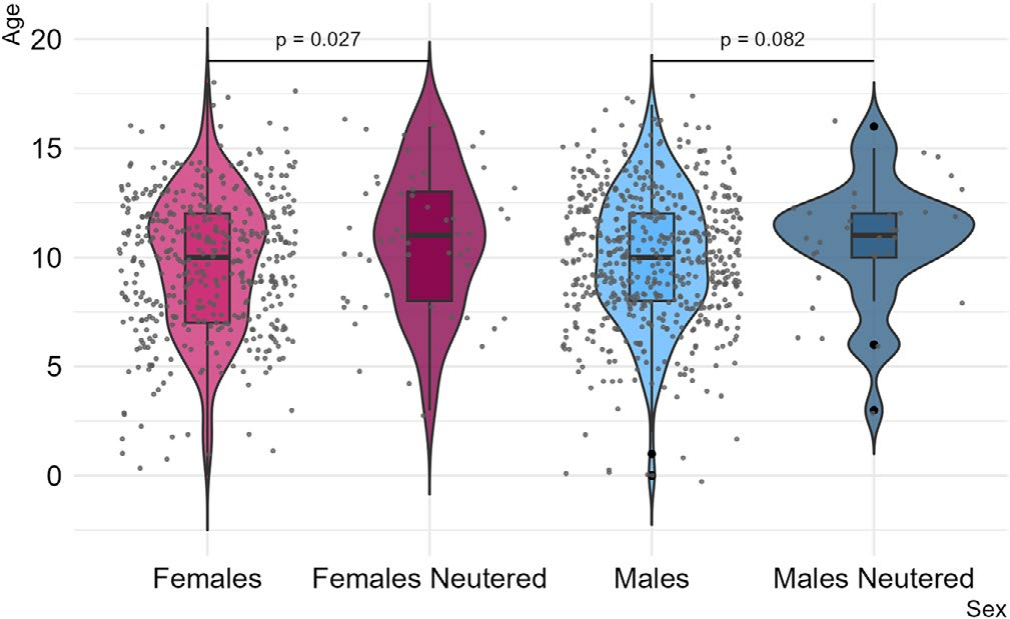
Violin plots showing the age distribution of melanocytic tumours' diagnosis between neutered and non‐neutered female and male dogs. Dots are horizontally jittered for visibility; horizontal position is arbitrary.

In dogs, no significant differences in the mean age were observed between the sexes (females: 9.9 years, SD = 3.1; males: 10.0 years, SD = 3.0) (Table S2). However, a noteworthy difference emerged when comparing neutered dogs to intact dogs: neutered dogs had a significantly higher mean age at diagnosis (10.8 years, SD = 3.0) compared to intact dogs (9.9 years, SD = 3.0) (*p* = 0.011) (Table S2). This age difference was particularly pronounced among female dogs, where neutered females had a higher mean age (10.8 years, SD = 3.2) than their intact counterparts (9.8 years, SD = 3.1) (*p* = 0.027). In contrast, no statistically significant differences were found between neutered and intact male dogs (*p* = 0.082) (Figure 1; Table S2).

When looking at the breed distribution of dogs with MTs (Table 3), mixed‐breed dogs were the most affected, comprising 26.7% of cases (*n* = 318). This was followed by Labrador Retrievers (9.8%, *n* = 117), Shih Tzus (8.7%, *n* = 103), Yorkshire Terriers (5.0%, *n* = 59), and Golden Retrievers (3.4%, *n* = 41).

**TABLE 3:** Breed distribution, mean age, incidence risk (IR) per 100 000 dogs and relative risk (RR) analysis.

Morphology/breed (5 most frequent)	*N* (%)	Mean age (SD)	IR	95% CI	RR	95% CI	*p*
All melanocytic tumours							
Mixed‐breed	340 (28.4)	10.6 (3.0)	8.3	7.42–9.19	ref	—	—
Labrador Retriever	126 (10.5)	9.9 (2.7)	20	16.48–23‐45	2.4	1.95–2.94	< 0.001
Yorkshire Terrier	66 (5.5)	10.8 (2.8)	17.9	13.56–22.19	2.15	1.65–2.80	< 0.001
Golden Retriever	44 (3.7)	10.5 (2.9)	53.1	37.40–68.77	6.4	4.66–8.74	< 0.001
Shar‐Pei	35 (2.9)	9 (2.9)	118	78.9–157.2	14.2	10.03–20.12	< 0.001
German Shepherd	34 (2.8)	8.8 (2.7)	7.9	5.26–1.58	0.95	0.67–1.35	0.793
Melanomas, NOS							
Mixed‐breed	169 (37.3)	11 (3.0)	4.1	3.51–4.75	ref	—	—
Labrador Retriever	63 (13.9)	10.1 (2.8)	10	7.52–12.45	2.4	1.81–3.23	< 0.001
Yorkshire Terrier	29 (6.4)	11.6 (2.8)	7.9	5.00–10.72	1.9	1.28–2.82	0.001
Golden Retriever	26 (5.7)	11.6 (2.4)	31.4	19.31–43.43	7.6	5.03–11.48	< 0.001
Shar‐Pei	12 (2.6)	8.8 (3.4)	40.5	17.58–63.38	9.8	5.46–17.60	< 0.001
German Shepherd	11 (2.4)	9.6 (1.6)	2.6	1.05–4.08	0.6	0.34–1.14	0.126
Amelanotic melanomas							
Mixed‐breed	44 (32.6)	12.1 (3.4)	1.1	0.76–1.39	ref	—	—
Labrador Retriever	24 (17.8)	11.1 (2.5)	3.8	2.28–5.33	3.5	2.15–5.82	< 0.001
Golden Retriever	7 (5.2)	11.0 (1.2)	8.4	2.19–14.70	7.9	3.54–17.44	< 0.001
Yorkshire Terrier	6 (4.4)	12.0 (2.3)	1.6	0.32–2.93	1.5	0.64–3.55	0.342
Poodle	6 (4.4)	13.0 (4.0)	3.6	0.71–6.41	3.3	1.41–7.77	0.006
German Shepherd	5 (3.7)	7.3 (1.7)	1.2	0.14–2.19	1.1	0.43–2.73	0.864
Melanocytomas							
Mixed‐breed	124 (31.6)	9.5 (2.5)	3	2.50–3.56	ref	—	—
Labrador Retriever	38 (9.7)	9.1 (2.5)	6	4.11–7.94	2	1.38–2.86	< 0.001
Yorkshire Terrier	31 (7.9)	9.6 (2.6)	8.4	5.44–11.35	2.8	1.87–4.11	< 0.001
Shar‐pei	20 (5.1)	8.6 (2.5)	67.5	37.90–97.04	22.3	13.88–35.70	< 0.001
German Shepherd	18 (4.6)	8.6 (3.2)	4.2	2.26–6.13	1.4	0.84–2.27	0.197
Portuguese Pointer	5 (1.3)	9.8 (2.2)	0.4	0.05–0.83	0.1	0.06–0.36	< 0.001

Age analysis within specific breeds revealed that Yorkshire Terriers had the highest mean age at diagnosis (10.8 years, SD = 2.8), while Rhodesian Ridgebacks were diagnosed at the youngest mean age (7.4 years, SD = 2.2) (Table 3).

**FIGURE 2:**
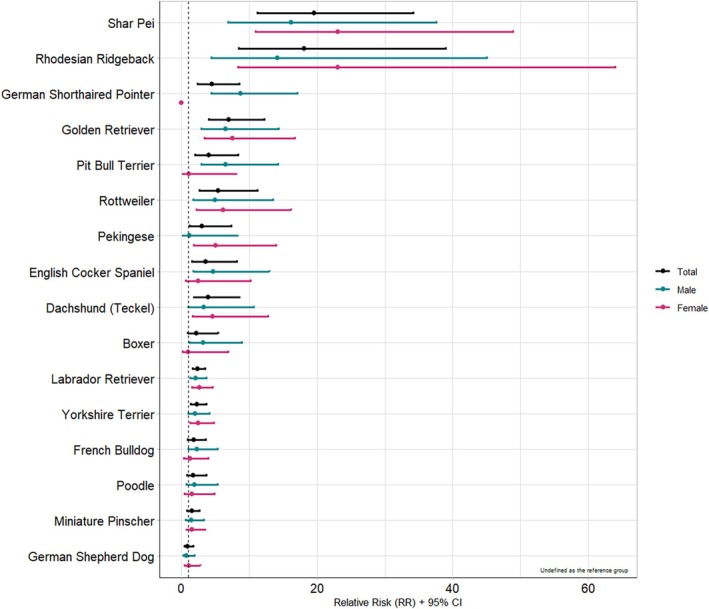
Relative risk (RR) estimates with 95% confidence intervals for each breed, stratified by sex for all melanocytic tumours taking the mixed breed as the reference group.

When it comes to relative risk (RR) (Figure 2), Shar‐Peis had the highest RR for developing MTs (RR = 14.2, *p* < 0.001) compared to mixed‐breed dogs, followed closely by Rhodesian Ridgebacks (RR = 12.2, *p* < 0.001) and Golden Retrievers (RR = 6.4, *p* < 0.001).

However, because Rhodesian Ridgebacks and Shar‐Peis are less common in Portugal than Golden Retrievers, the latter breed is considered to have the highest RR of developing MTs in the country. Golden Retrievers showed an incidence rate (IR) of 67.6 and a RR of 6.9 compared to mixed‐breed dogs (*p* < 0.001). They were particularly prone to melanomas (IR = 31.4; RR = 7.6, *p* < 0.001) and amelanotic melanomas (IR = 8.4; RR = 7.9, *p* < 0.001), with a notable susceptibility to oral melanoma (IR = 28.9; RR = 12.8, *p* < 0.001).

The Shar‐Pei also had a notably high IR for melanoma (IR = 40.5; RR = 9.8, *p* < 0.001). After Golden Retrievers, Labrador Retrievers showed the highest predisposition to amelanotic melanomas (IR = 3.8; RR = 3.5, *p* < 0.001). Finally, the Shar‐Pei exhibited the highest risk of developing melanocytomas (IR = 67.5; RR = 22.3, *p* < 0.001), followed by the Yorkshire Terrier (IR = 8.4; RR = 2.8, *p* < 0.001) (Table 3).

### 3.4 Comparative Analysis

Melanoma, NOS is the most common type of MT across all species, with the highest frequency in humans (95.7%, *n* = 17 532) and cats (81.3%, *n* = 78). Although still common in dogs, it appears at a lower percentage (44.5%, *n* = 487). Melanocytomas, on the other hand, are notably frequent in dogs (42.1%, *n* = 460) but rare in both cats (11.5%, *n* = 11) and humans (1.8%, *n* = 338). As for amelanotic melanomas, dogs are the most affected (13.35%, *n* = 145), with lower occurrences in cats (7.3%, *n* = 7) and humans (0.14%, *n* = 25).

Looking at annual incidence rates (IR), dogs (16.1 per 100 000 dogs) show double that of humans' IR (8.1 cases per 100 000) for all melanocytic tumours, while cats have a slightly lower IR (6.3 per 100 000) than humans. However, humans have a higher IR for melanomas (7.71 per 100 000) compared to both dogs (5.96 per 100 000) and cats (5.41 per 100 000), which have nearly identical IRs. Dogs show a much higher IR for amelanotic melanomas (1.82 per 100 000) than humans (0.01 per 100 000) and cats (0.28 per 100 000). When it comes to benign melanocytic tumours, dogs have a significantly higher IR (8.29 per 100 000) compared to both humans (0.37 per 100 000) and cats (0.57 per 100 000).

**FIGURE 3:**
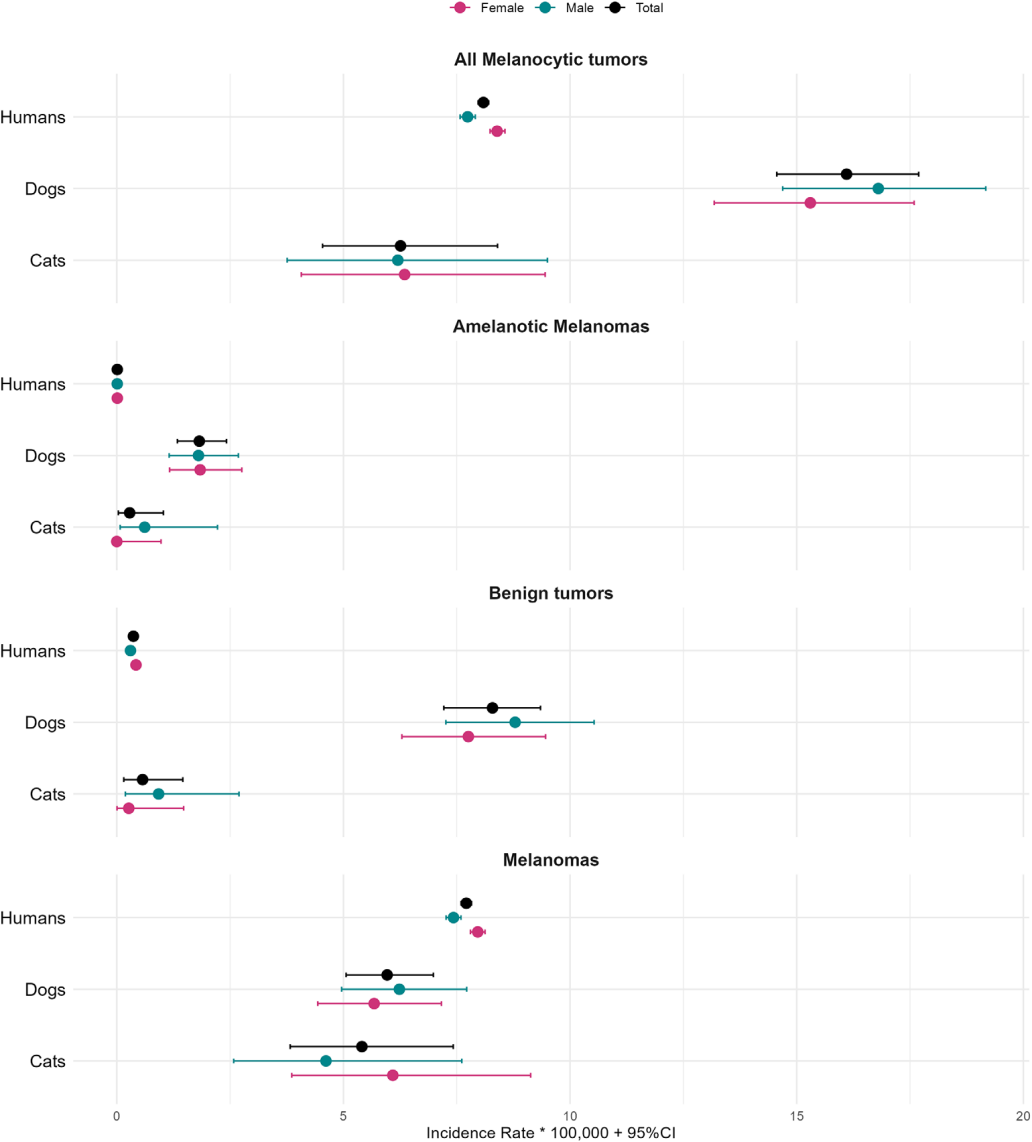
Incidence rates of MTs in general, amelanotic melanomas, benign MTs and melanomas in humans, dogs and cats.

The full IR for all MTs across humans, dogs, and cats is detailed in Figure 3 and Table S3. Overall, dogs show the highest incidence for all MTs, particularly benign ones like melanocytomas. They also develop more amelanotic melanomas than humans and cats, though the differences are not as pronounced as with all melanocytic and benign tumours. Last, melanomas appear slightly more prevalent in humans than in dogs and cats, as highlighted in Figure 3.

### 3.5 Age

**FIGURE 4:**
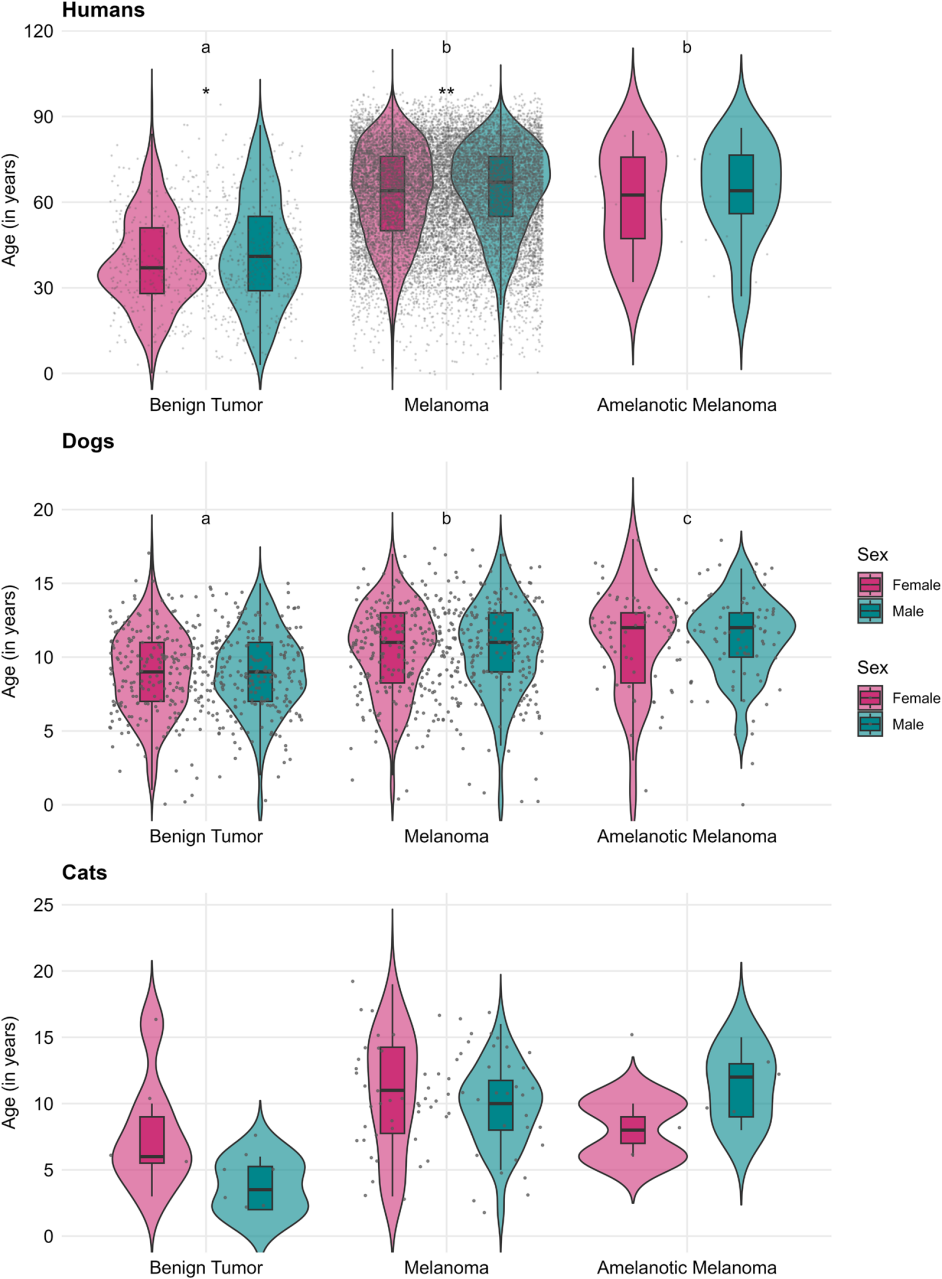
Age distribution of MTs in humans, cats and dogs. The mean age of incidence for all MTs, as well as for each specific type of tumour, along with the age differences between males and females across the three species, is detailed in Table S4. *Note*: *p < 0.05; **p < 0.01. Asterisks denote sex differences within each morphology and species based on two‐sample *t*‐tests. Different letters above the boxes indicate statistically distinct morphology groups (one‐way ANOVA followed by Tukey's HSD, α = 0.05); groups sharing the same letter do not differ significantly.

The mean age of incidence for MTs varies significantly across species: humans develop these tumours at an average age of 62.1 years (SD = 17.2), dogs at 10 years (SD = 3.1) and cats at 9.9 years (SD = 4.0) (Figure 4).

In humans, both men and women tend to develop melanoma and amelanotic melanoma later in life compared to benign MTs (Figure 4). Similarly, in dogs, both males and females generally develop all types of MTs at a later age, with melanomas and amelanotic melanomas appearing somewhat later than benign tumours (Figure 4).

For cats, female cats develop melanomas later than males, while male cats tend to develop amelanotic melanomas at a later age than their female counterparts (Figure 4). Interestingly, male cats develop benign MTs at a much earlier age than females, and this early onset is even more striking when compared to dogs and humans, who typically develop these tumours at older ages (Figure 4). However, these findings should be regarded with caution, as the number of cats in this study is very small: five males and eight females.

**FIGURE 5:**
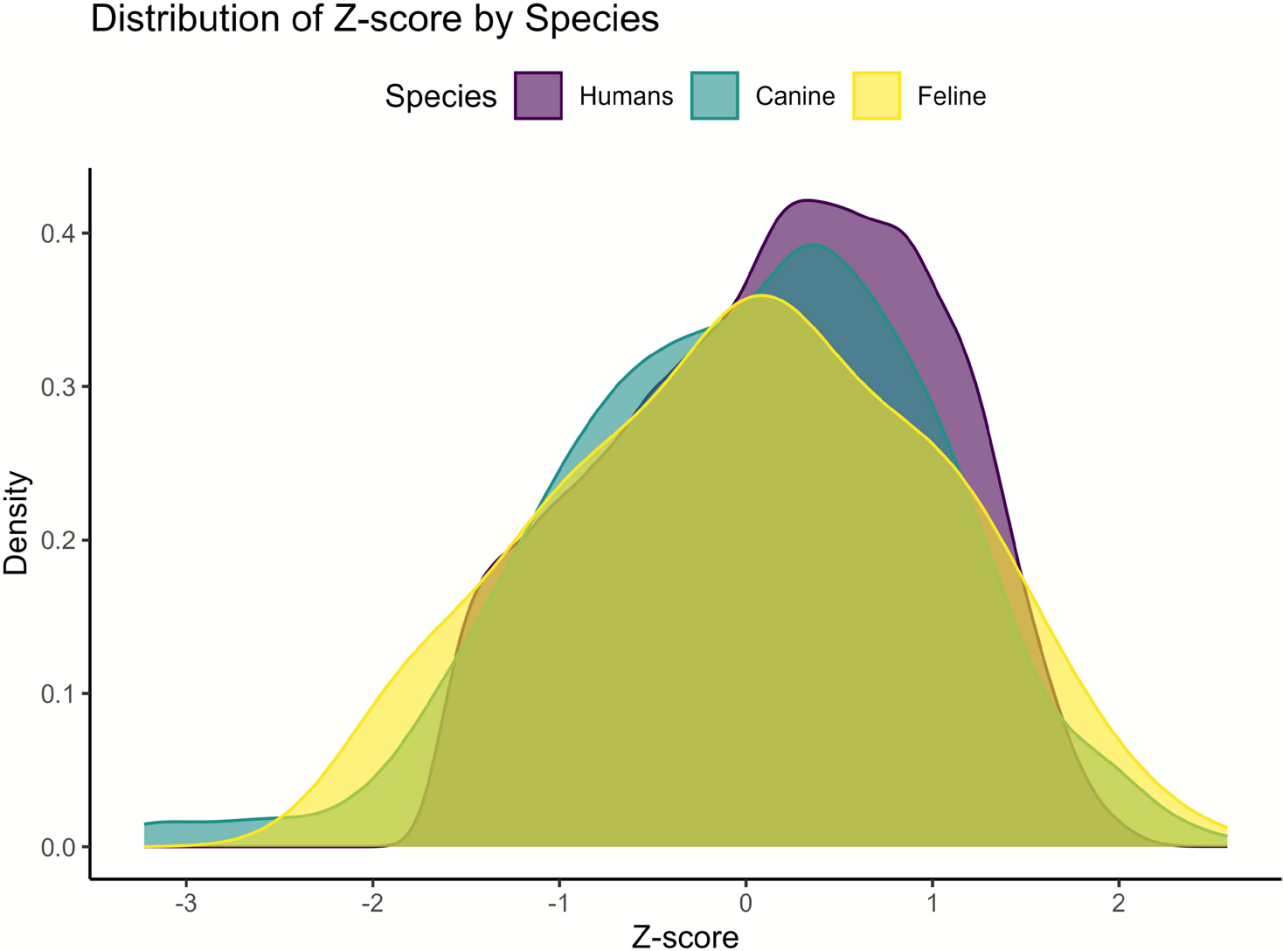
Density plots comparing the *Z*‐score of the age at diagnosis of all melanocytic tumours between humans, dogs and cats.

Figure 5 analysis reflects a very similar age distribution between the three species. However, the Kolmogorov–Smirnov (KS) test indicated a highly significant difference between humans and canines (*D* = 0.125, *p* < 0.001). Similarly, a significant difference was observed between humans and felines (*D* = 0.141, *p* = 0.044), though the magnitude of the difference was less pronounced. In contrast, no significant difference was detected between canines and felines (*D* = 0.124, *p* = 0.130), indicating that their distributions are likely similar.

### 3.6 Geographic Distribution

The pattern of the smoothed standardised incidence ratios (SIR) for MTs in both humans and dogs exhibits significant clustering. However, the degree of spatial autocorrelation is substantially higher among humans (Moran's *I* = 0.471, *p* < 0.001) compared to dogs (Moran's *I* = 0.212, *p* < 0.001).

**FIGURE 6:**
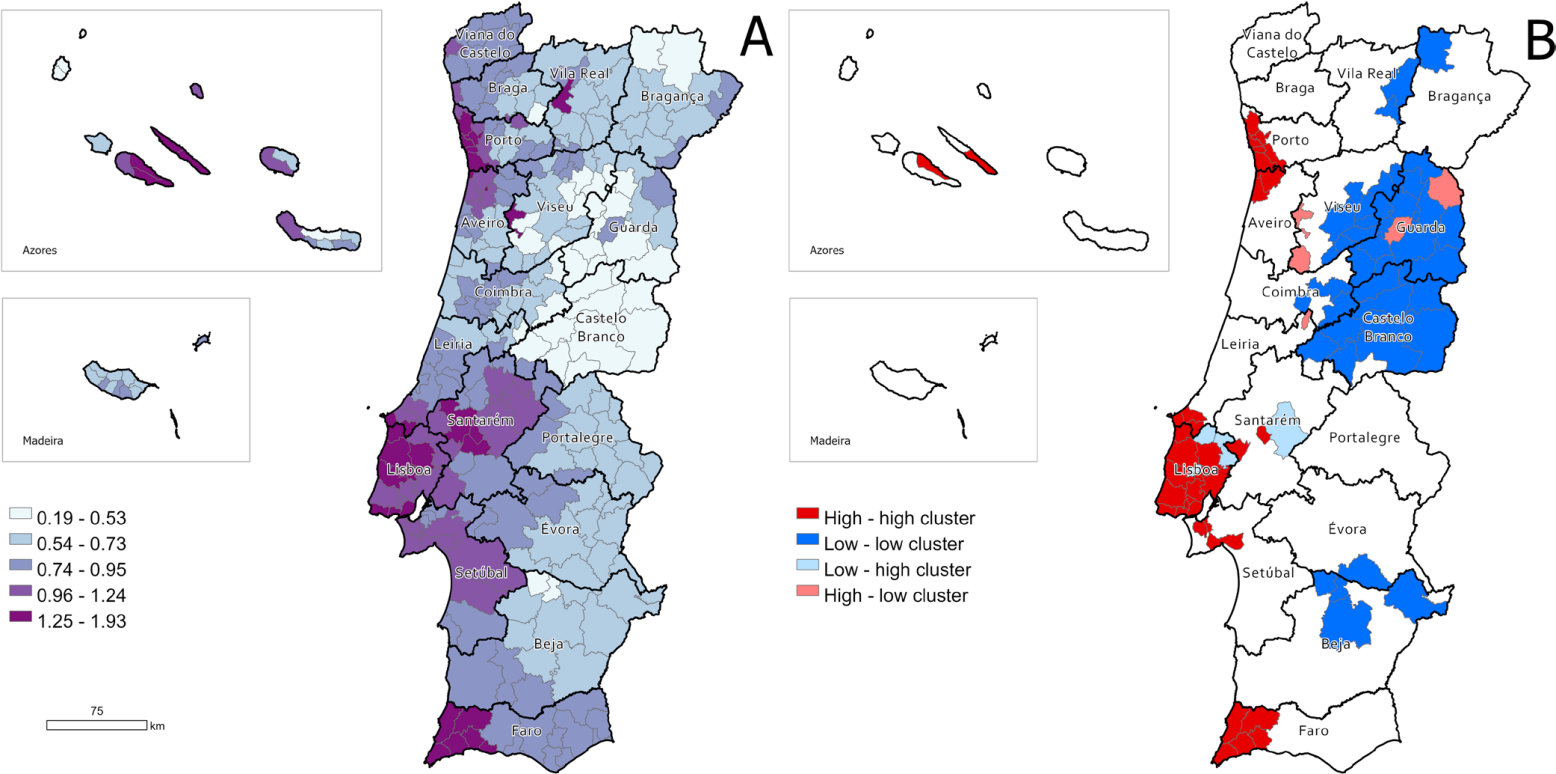
Geographical distribution of the standardised incidence ratios (SIR) of melanocytic tumours among humans. (A) SIR according to municipality. (B) LISA cluster map highlighting geographical clusters and outliers.

The smoothed SIR and LISA clusters for MTs in humans are shown in Figure 6. The smoothed SIR reveals a clear longitudinal gradient, with ratios increasing from inland areas toward the coast. Higher ratios are concentrated in the coastal and highly urbanised metropolitan areas of Porto and Lisbon, and also in the western part of Faro district and the Central islands of the Azores archipelago, where several high–high clusters were identified. In contrast, lower ratios are predominantly found in inland municipalities, particularly in the Guarda and Castelo Branco districts, which feature a large low–low cluster.

**FIGURE 7:**
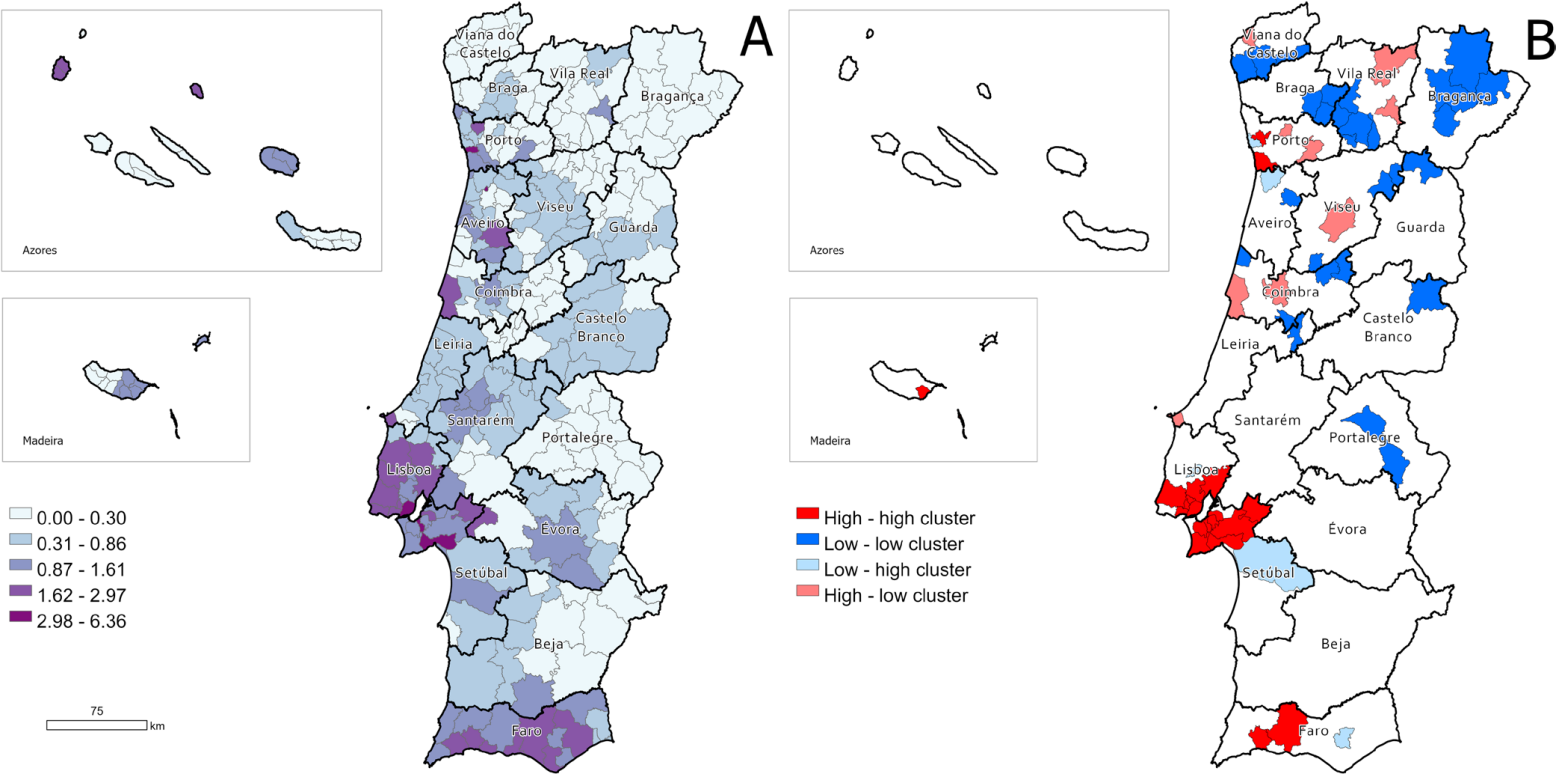
Geographical distribution of the standardised incidence ratios (SIR) of MTs among dogs. (A) SIR, according to the municipality. (B) LISA cluster map highlighting geographical clusters and outliers.

The smoothed SIR and LISA clusters for MTs in dogs are shown in Figure 7. In comparison to the geography of human MTs, these maps reveal less distinct patterns. Nevertheless, similar to humans, higher ratios are observed along the coast, particularly in the districts of Porto, Lisboa, Setúbal and Faro, where high‐high clusters (i.e., areas with a high incidence of MTs that are surrounded by neighbouring areas also exhibiting high incidence rates) were identified. Conversely, lower ratios tend to be concentrated in the North and Central regions and inland areas of the country, with several low‐low clusters (i.e., areas with low incidence rates that are surrounded by neighbouring areas with similarly low rates) scattered through these geographical areas.

**FIGURE 8:**
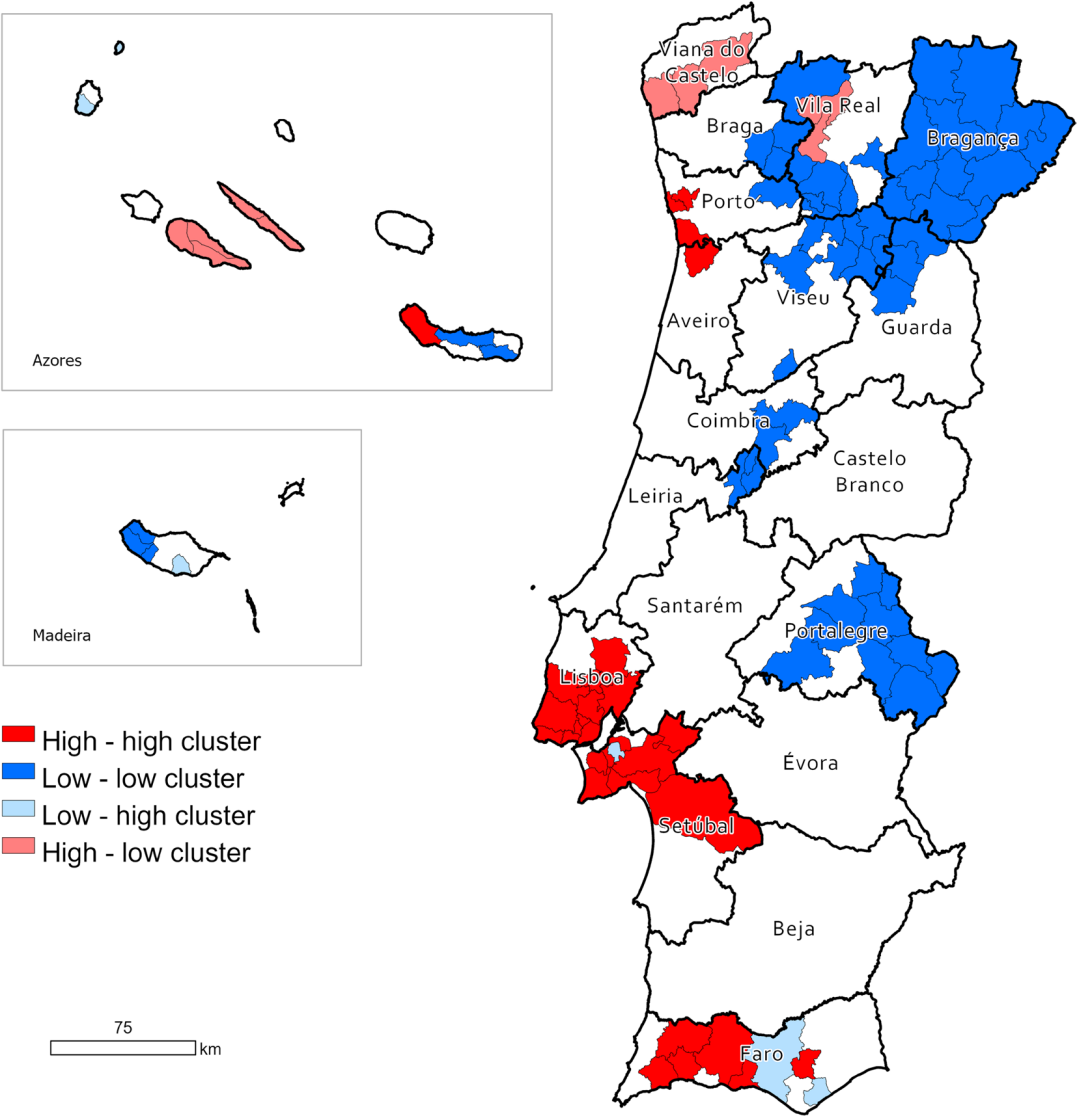
BLISA cluster map highlighting geographical clusters and outliers. The map identifies statistically significant spatial clusters and outliers using the local Moran's *I* statistic. High–high clusters represent areas with high incidence rates of melanocytic tumours surrounded by neighbouring areas also with high rates, indicating potential hotspots of elevated risk; low–low clusters represent areas with low incidence surrounded by neighbouring areas also exhibiting low rates, suggesting regions of consistently low risk (coldspots); high–low and low–high areas are spatial outliers, where an area with a high (or low) incidence is surrounded by areas with the opposite trend.

Furthermore, the Bivariate Moran Local Index (BLISA) was applied to identify areas with simultaneous high or low ratios of MTs in both humans and dogs. A statistically significant spatial correlation was observed, with a BLISA value of 0.345 (*p* < 0.001). As illustrated in Figure 8, the BLISA analysis identified clusters of simultaneous high ratios of MTs in both humans and dogs across the Lisbon metropolitan area and the western part of Faro district, and also, to a lesser extent, the Porto metropolitan area. On the other hand, clusters of simultaneous humans' and dogs' low ratios of MTs spread across inner Portugal, with larger ones almost covering the entire northeastern Portugal and the district of Portalegre, and minor ones located in the inner areas of the Viseu, Coimbra, and Leiria districts.

## 4 Discussion

MTs in dogs, cats and humans share numerous similarities, and all three species are exposed to similar environments and potential carcinogens, making companion animals valuable models for comparative oncology [4, 5, 7, 8, 12]. Despite this, no comprehensive epidemiological studies have yet compared the incidence and relative risks of MTs and their geographical distribution across these three species in Portugal. This study sought to address this gap and aimed to identify which types of MTs are more common in each species, considering factors such as sex and the median age of tumour development. The IRs were calculated for all three species. Additionally, the RR for developing MTs—melanocytoma, melanoma and amelanotic melanoma—was calculated for different dog breeds. However, in cats, the number of breeds and cases registered per breed was too low, not enabling the calculation of the RR for cats without having bias.

In this study, humans, dogs, and cats exhibited a strikingly similar age distribution for the development of MTs, although dogs, but also cats, appeared to develop these neoplasms at an earlier age. Interestingly, there were no significant differences in the mean age of tumour development between the sexes, though neutered dogs, especially females, showed a higher average age at diagnosis. This finding could be influenced by a lack of registered data regarding the neutered status of animals in the SIAC system, coupled with the cultural tendency to avoid neutering male dogs, which may amplify the observed effect. However, the authors found these results interesting, and more investigation regarding this is needed.

In humans, oestrogens and oestrogen receptors are believed to influence melanoma development, though the relationship is debated [28]. Reduced ovarian hormone exposure may lower melanoma risk [29], and Mai et al. found higher incidence in women with older age at first birth, earlier menarche and increased age [30]. Joosse et al. found that women with cutaneous melanoma generally have a better prognosis than men, both pre‐ and postmenopausal [31]. However, Lasithiotakis et al. showed that after age 65, women's survival advantage diminishes, with higher RR of mortality [32]. Enninga et al. found that women with regional melanoma had lower mortality risk than men of the same age [33]. These findings highlight the complex interplay of age, sex hormones and prognosis in melanoma, underscoring the importance of these factors in both human and comparative oncology.

In metastatic melanoma, there was no significant sex or age effect on survival, suggesting sex impacts outcomes mainly in early‐stage disease [33]. The influence of age at menarche and menopause on melanoma risk remains unclear, with mixed findings [28]. While oestrogen's role in dogs with MTs has not been studied, our results suggest a difference between neutered and non‐neutered animals, indicating that reproductive hormones may play a role. Additionally, MTs were more common in older dogs and cats, consistent with previous studies [34].

In this study, MTs were more prevalent in women; however, this may not be caused only by biological reasons but also by socio‐cultural factors, as women may have more skin cancer awareness than men and, therefore, have a higher detection of these neoplasms, which could account for the difference in results between women and men.

Additionally, it should be noted that in humans, only excised tumours that were sent for histopathology were included in this study, as some lesions that do not present suspicious macroscopic features of malignancy are frequently removed by laser or similar methods and not sent for histological examination. On the other hand, in animals, treatment is predominantly surgical, regardless of biological behaviour, and the tumour is always sent for histopathological examination. Therefore, this may lead to bias, as there is a possibility of an underrepresentation of benign tumours and of an overestimation of malignant tumours in humans, and when comparing humans' and dogs' results, this may explain the higher incidence of melanocytomas in the latter. All the tumours used in this study were codified based on the International Classification of Diseases for Oncology (ICD‐O) for humans and on the Veterinary International Classification of Diseases for Oncology (Vet‐ICD‐O) for dogs and cats in the Portuguese Human Cancer Registry (RON) and in the Portuguese Veterinary Cancer Registry (Vet‐Onconet), respectively, and have a histopathological result associated; therefore, malignant and benign tumours in all species are grouped accordingly.

In Portugal, the most prevalent type of MTs in humans and cats was Melanoma, NOS, which is also common in dogs, though at a lower rate. Melanocytomas were more common in dogs but rare in humans and cats, while amelanotic melanomas were much more frequent in dogs than in either cats or humans. Other studies in cats and dogs have similarly shown a higher prevalence of melanomas compared to melanocytomas, especially in cats [34]. Dogs have many benign tumours, such as melanocytomas; however, the reason why this happens has not been discovered yet, and more research is needed [35].

Regarding age analyses in cats, it is important to notice that intraocular tumours in these animals often grow slowly, and it is common that enucleation is delayed until discomfort or glaucoma develops, which may influence the mean age at diagnosis, and possibly leading to bias. Also, it is important to notice that ocular and eyelid tumours are two separate groups, as the eyelid tumours are part of the skin group (C44) and the ocular tumours are part of the eyes and adnexa group (C69.‐), so they were not analysed together neither in dogs, cats or humans.

Regarding dogs' breed, MTs were mostly diagnosed in mixed‐breed dogs, followed by Labrador Retrievers, Yorkshire Terriers, and Golden Retrievers, which were also overrepresented breeds in other studies [34].

In accordance with other reports [15, 34, 36, 37, 38], the most frequent topography for the development of MTs in humans, dogs, and cats was the skin. However, we acknowledge that differentiating between melanocytomas and well‐differentiated melanomas can be challenging, and borderline cases may be interpreted differently among pathologists, introducing potential inter‐study variability. Furthermore, it is well recognised that melanocytic tumours arising from specific sites such as the nail bed, mucocutaneous junctions, or oral mucosa often display a more aggressive biological behaviour compared to those originating from haired skin, which are frequently benign. In the present study, all cutaneous tumours were classified based on their morphology and degree of malignancy according to the Veterinary International Classification of Diseases for Oncology (Vet‐ICD‐O). Therefore, although all lesions were included under the general category of skin tumours, they were further separated and analysed considering their histopathological characteristics and malignancy grade, ensuring that these biological differences were taken into account.

Annual IR was very similar for all MTs in humans and dogs, but smaller in cats. The Golden Retrievers had the highest IR to MTs overall, and particularly to oral melanoma; Labrador Retrievers to amelanotic melanomas; and Shar‐pei to melanocytomas.

In addition, recent studies have reported that the Rhodesian Ridgeback—a breed with the highest relative risk (RR) for MTs in our study—appears predisposed to MTs, even though it is relatively uncommon in Portugal [34]. Although no previous reports have indicated a predisposition for MTs in the Shar‐Pei, our study found that this breed had the second highest RR. The literature indicates that breeds with heavily pigmented skin and coats are more susceptible to developing MTs [3, 34]. While Shar‐Peis do not have highly pigmented skin or coats, their oral mucosa is heavily pigmented, and breeds with a highly pigmented mucosa appear to be more prone to oral melanoma. Interestingly, our study also showed that Shar‐Peis had the highest IR for melanocytomas, which typically occur on the skin. Therefore, further studies involving a larger sample of Shar‐Peis are needed to determine whether this breed is indeed predisposed to MTs.

Regarding the geographical distribution of MTs in humans and dogs, both species exhibit significant clustering; however, the degree of spatial correlation is higher in humans than in dogs, and these animals' melanocytic tumours also reveal less distinct patterns than humans.

Nonetheless, similarly to humans, higher ratios of MTs in dogs are observed along the coast of Portugal, especially in districts such as Porto, Lisboa, Setúbal and Faro. Additionally, low ratio clusters of MTs in both humans and dogs were observed across inner Portugal, particularly in the northeastern area and the district of Portalegre, and minor clusters were also observed in the inner areas of Viseu, Leiria and Coimbra districts. There are many possible factors that may cause this geographic distribution of MTs, as higher pollution and population density in urban areas can lead to more cancer cases in these regions. Looking at population density in particular, the spatial clustering of dog registrations in coastal urban areas such as Lisbon and Porto mirrors broader demographic and socioeconomic patterns in Portugal [39]. Urban centers, characterised by higher population density, show a preference for smaller and international breeds, while rural areas favour Portuguese native breeds [39]. These differences likely reflect lifestyle and housing conditions, as well as disparities in access to veterinary care. Urban populations benefit from more available routine services, whereas rural and economically disadvantaged regions often face infrastructural, financial and educational barriers [39]. Such inequalities may influence both dog distribution and health outcomes. Moreover, the inverse relationship between income and number of dogs per household highlights the role of economic capacity in pet ownership [39]. Overall, these findings emphasise the need to consider socioeconomic and environmental determinants when interpreting spatial patterns, and future studies should explore breed‐based standardisation and urbanisation overlays to refine this analysis. Additionally, sun exposure is also higher in the south of Portugal, especially in the Faro district, which can also lead to more MTs cases in humans; even though in dogs the development of MTs is not related to sun exposure according to what we know until now, more research in this field is needed to confirm that this factor really does not have an influence on the development of MTs in dogs.

These results align with other comparative studies done in the Porto region regarding breast cancer and non‐Hodgkin's lymphoma in humans and dogs that demonstrated a close geographical association between the two species at the municipality level [13, 14].

These findings underscore the complex and cross‐species nature of MTs and emphasise the potential value of dogs as sentinels for research studies in human oncological epidemiology and for comparative oncology studies to improve understanding across species.

## 5 Limitations

While the study offers significant insights, several limitations must be acknowledged. First, the temporal data for human and animal cases do not align. Nevertheless, the findings support continued investigation into tumours in dogs and cats until a temporal window comparable to—or even preceding—that of human data is achieved. Additionally, the absence of data from the Vila Real region (northeast Portugal) is a notable limitation, as evidenced by the maps indicating low clusters in that area. Moreover, as is common in population‐based animal studies, there is inherent underrepresentation in both the numerator (number of cases) and the denominator (population at risk). Despite these issues, the database is robust, boasting strong external representation and setting a new benchmark in veterinary medicine in Portugal. Ongoing efforts to expand and integrate data are expected to enhance its coverage and reliability over time.

At the time of this study, breed‐standardised geographical analyses were not possible due to the lack of available data, so the performed analyses were age‐standardised. Now that breed distribution data have recently become available (Pinello et al.), future research could consider breed‐standardised analyses to further refine spatial patterns.

Additionally, it should be noted that in humans, only excised tumours sent for histopathology were included, as lesions without suspicious macroscopic features are often removed by laser or similar methods and not examined. In animals, treatment is usually surgical and tumours are always analysed, which may introduce bias—underrepresenting benign tumours and overestimating malignant ones in humans, possibly explaining the higher incidence of melanocytomas in dogs.

## 6 Conclusion

This is the first epidemiological study to compare the incidence of melanocytic tumours in dogs and cats with humans, shedding light on the shared characteristics between these tumours in the three species, highlighting the potential of cross‐species oncological studies and the importance of comparative oncology. It also shows the most predisposed breeds of dogs to these types of tumours and highlights the fact that neutered dogs seem to have a protective factor for the development of these tumours, which needs more research on why this may happen. Finally, regarding the geographical distribution, both humans and dogs showed high and low clusters of melanocytic tumours in the same regions of Portugal, enhancing the evidence of shared risks and the role as sentinels for cancer developing.

## Author Contributions

**C.A.P.:** conceptualization, data curation, formal analysis, investigation, methodology, writing – original draft, writing – review and editing. **A.I.R.:** data curation, formal analysis, investigation, validation, visualisation. **J.N.‐R.:** validation, visualisation. **C.A.P.d.S.:** validation, visualisation, writing – review and editing. **K.P.:** conceptualization, investigation, data curation, formal analysis, methodology, validation, visualisation, writing – original draft, writing – review and editing. **A.A.F.S.:** conceptualization, investigation, methodology, supervision, writing – original draft, writing – review and editing.

## Funding

Catarina Alves Pinto was supported by grant 2023.04484.BD from Fundação Para a Ciência e a Tecnologia (FCT), Portugal. Vet‐OncoNet received support from the ICBAS—School of Medicine and Biomedical Sciences from the University of Porto. Ana Isabel Ribeiro was supported by National Funds through FCT, under the “Stimulus of Scientific Employment – Individual Support” Programme within the contract CEECIND/02386/2018 (https://doi.org/10.54499/CEECIND/02386/2018/CP1538/CT0001). This work was supported by FCT—Fundação Para a Ciência e Tecnologia, I.P. through the projects with references UIDB/04750/2020 and LA/P/0064/2020 and DOI identifiers https://doi.org/10.54499/UIDB/04750/2020 and https://doi.org/10.54499/LA/P/0064/2020. This research also received support from the Centre of Studies in Geography and Spatial Planning (CEGOT), funded by national funds through the Foundation for Science and Technology (FCT) under the reference UIDB/04084/2025, and through FCT funding attributed to the Research Center of IPO‐Porto under the reference UID/00776/2025 and DOI identifier https://doi.org/10.54499/UID/00776/2025.

## Ethics Statement

This study was approved by the Animal Welfare Ethics Committee (ORBEA) of the ICBAS—School of Medicine and Biomedical Sciences, University of Porto (P394/2021/ORBEA). The use of human data was approved by the Ethics Committee of the Portuguese Institute of Oncology (IPO) of Porto with the reference number CES 039/024.

## Consent

Written informed consent for participation was not required from the patients or their legal guardians/next of kin in accordance with the national legislation and institutional requirements, and the study was conducted in accordance with these same legislations and requirements.

## Conflicts of Interest

The authors declare no conflicts of interest.

## Supporting information


**Data S1:** vco70031‐sup‐0001‐Supinfo.docx.

## Data Availability

The original data presented in the study is included in the article and Supporting Information. Further inquiries can be directed to the corresponding author.
