# Supplementary material for: A Comparative Study of Melanocytic Tumours: Linking Portuguese Dogs and Cats to Human Cases
Source: Vet Comp Oncol. 2025 Dec 10;24(1):105–20. doi: 10.1111/vco.70031 (PMC12875750; doi:10.1111/vco.70031)
Supplement: Supplementary file 1 — Data S1: vco70031‐sup‐0001‐Supinfo.docx. [file VCO-24-105-s001.docx]

**Supplementary Material**

**Table S1:** Classification of melanocytic tumor morphologies included in each group, along with their respective ICD-O-3.2 codes.

| **Morphology** |  |  |  |  |  |  |  |
| --- | --- | --- | --- | --- | --- | --- | --- |
|  | **Total** | | **Females** | | **Males** | |  |
|  | **n** | **%** | **n** | **%** | **n** | **%** |  |
| **Melanoma** | **17469** | **100,0** | **9529** | **54,6** | **7940** | **45,5** |  |
| Low cumulative sun damage melanoma_(8743/3)_ | 4986 | 27,2 | 2654 | 53,2 | 2332 | 46,8 |  |
| Nodular melanoma _(8721/3)_ | 1553 | 8,5 | 745 | 48,0 | 808 | 52,0 |  |
| Lentigo maligna melanoma _(8742/3)_ | 1438 | 7,9 | 832 | 57,9 | 606 | 42,1 |  |
| Acral melanoma _(8744/3)_ | | 582 | 3,2 | 359 | 61,7 | 223 | 38,3 |
| Malignant melanoma in junctional nevus _(8740/3)_ | 161 | 0,9 | 98 | 60,9 | 63 | 39,1 |  |
| Malignant Spitz tumor _(8770/3)_ | 88 | 0,5 | 52 | 59,1 | 36 | 40,9 |  |
| Desmoplastic melanoma, NOS _(8745/3)_ | 71 | 0,4 | 40 | 56,3 | 31 | 43,7 |  |
| Epithelioid cell melanoma _(8771/3)_ | 64 | 0,4 | 27 | 42,2 | 37 | 57,8 |  |
| Malignant melanoma arising in giant congenital nevus _(8761/3)_ | 64 | 0,4 | 33 | 51,6 | 31 | 48,4 |  |
| Blue nevus, malignant _(8780/3)_ | 63 | 0,3 | 32 | 50,8 | 31 | 49,2 |  |
| Spindle cell melanoma, NOS _(8772/3)_ | 54 | 0,3 | 27 | 50,0 | 27 | 50,0 |  |
| Malignant melanoma in precancerous melanosis _(8761/3)_ | 35 | 0,2 | 21 | 60,0 | 14 | 40,0 |  |
| Mucosal lentiginous melanoma _(8746/3)_ | 28 | 0,2 | 21 | 75,0 | 7 | 25,0 |  |
| Malignant melanoma, regressing _(8723/3)_ | 17 | 0,1 | 9 | 52,9 | 8 | 47,1 |  |
| Spindle cell melanoma, type B _(8774/3)_ | 6 | 0,0 | 1 | 16,7 | 5 | 83,3 |  |
| Spindle cell melanoma, type A _(8773/3)_ | 3 | 0,0 | 1 | 33,3 | 2 | 66,7 |  |
| **Melanocytoma** | **830** | **100,0** | **507** | **61,1** | **323** | **38,9** |  |
| Compound nevus _(8760/0)_ | 429 | 2,3 | 266 | 62,0 | 163 | 38,0 |  |
| Dermal nevus _(8750/0)_ | 187 | 1,0 | 123 | 65,8 | 64 | 34,2 |  |
| Dysplastic nevus _(8727/0)_ | 147 | 0,8 | 83 | 56,5 | 64 | 43,5 |  |
| Meningeal melanocytosis _(8728/0)_ | 3 | 0,0 | 2 | 66,7 | 1 | 33,3 |  |
| Magnocellular nevus _(8726/0)_ | 1 | 0,0 | 1 | 100,0 |  | 0,0 |  |
| **Amelanotic Melanoma** |  |  |  |  |  |  |  |
| Amelanotic melanoma _(8730/3)_ | **25** | **100,0** | **14** | **56,0** | **11** | **44,0** |  |

**Table S2**: Differences between the mean age of development of melanocytic tumors in intact and neutered dogs

| **Sex** | **N** | **Mean** | **SD** | **Median** | **Minimum** | **Maximum** | **Total** |
| --- | --- | --- | --- | --- | --- | --- | --- |
| F (Total) | 589 | 9.9 | 3.1 | 10 | 0 | 18 | 494 |
| Intact Female | 525 | 9.8* | 3.1 | 10 | 0 | 18 | 440 |
| Neutered Female | 64 | 10.8* | 3.2 | 11 | 3 | 16 | 54 |
| M (Total) | 706 | 10.0 | 3.0 | 10 | 0 | 17 | 600 |
| Intact Male | 664 | 10.0 | 3.0 | 10 | 0 | 17 | 567 |
| Castrated Male | 42 | 10.8 | 2.8 | 11 | 3 | 16 | 33 |
| missings | 8 |  |  |  |  |  |  |
| Neutered (F+M) | 106 | 10.8 | 3.0 | 11 | 3 | 16 | 87 |
| Intact (F+M) | 1189 | 9.9 | 3.1 | 10 | 0 | 18 | 1007 |

**Statistically significative difference between intact and neutered females - p<0.05 (p=0.027)*

**Table S3:** Incidence Rates of melanocytic tumors in general, melanomas, amelanotic melanomas, and benign melanocytic tumors (and respective confidence intervals) in humans, dogs and cats.

| **Morphology/Species** | **IR** | **CI (Min-Max)** |
| --- | --- | --- |
|  |  |  |
| **All Melanocytic tumors** |  |  |
| Humans  Males  Females | 7,74  8,39 | 7,57 - 7,91  8,23 - 8,56 |
| Total | 8,09 | 7,97 - 8,21 |
| Dogs |  |  |
| Males | 16,80 | 14,69 - 19,17 |
| Females | 15,30 | 13,18 - 17,59 |
| Total | 16,10 | 14,56 - 17,69 |
| Cats |  |  |
| Males | 6,20 | 3,76 - 9,50 |
| Females | 6,35 | 4,07 - 9,45 |
| Total | 6,26 | 4,54 - 8,40 |
|  |  |  |
| **Melanomas** |  |  |
| Humans  Males  Females | 7,43  7,96 | 7,27 – 7,59  7,80 - 8,12 |
| Total | 7,71 | 7,60 – 7,83 |
| Dogs |  |  |
| Males | 6,23 | 4,96 - 7,72 |
| Females | 5,68 | 4,43 - 7,16 |
| Total | 5,96 | 5,06 - 6,98 |
| Cats |  |  |
| Males | 4,61 | 2,58 - 7,61 |
| Females | 6,09 | 3,86 - 9,13 |
| Total | 5,41 | 3,83 - 7,42 |
| **Amelanotic melanomas** |  |  |
| Humans  Males  Females | 0,01  0,01 | 0,01 - 0,02  0,01 - 0,02 |
| Total | 0,01 | 0,01 - 0,02 |
| Dogs |  |  |
| Males | 1,80 | 1,16 - 2,68 |
| Females | 1,84 | 1,17 - 2,76 |
| Total | 1,82 | 1,34 - 2,42 |
| Cats |  |  |
| Males | 0,62 | 0,07 - 2,22 |
| Females | 0,00 | 0,00 - 0,98 |
| Total | 0,28 | 0,03 - 1,03 |
| **Benign tumors** |  |  |
| Humans  Males  Females | 0,30  0,42 | 0,27-0,34  0,39 – 0,46 |
| Total | 0,37 | 0,34 – 0,39 |
| Dogs |  |  |
| Males | 8,79 | 7,26 - 10,53 |
| Females | 7,76 | 6,29 - 9,46 |
| Total | 8,29 | 7,22 – 9,35 |
| Cats |  |  |
| Males | 0,92 | 0,19 - 2,70 |
| Females | 0,26 | 0,01 – 1,48 |
| Total | 0,57 | 0,16 - 1,46 |

**Table S4.** Mean age of incidence of melanocytic tumors in general, melanoma, melanocytoma, and amelanotic melanoma in humans, dogs, and cats.

|  |  | **Humans** | | **Dogs** | | | **Cats** | | |
| --- | --- | --- | --- | --- | --- | --- | --- | --- | --- |
|  | **Mean age (SD)** | | | **Mean age (SD)** | | | **Mean age (SD)** | | |
|  | **Total** | **M** | **F** | **Total** | **M** | **F** | **Total** | **M** | **F** |
| All Melanocytic tumors | 62.1  (17.2) | 63.5 (16.4) | 61.0 (17.8) | 10.0  (3.1) | 10.0  (3.0) | 9.9  (3.2) | 9.9  (4.0) | 9.4  (3.4) | 10.3  (4.5) |
| Benign tumors | 40.5  (17.2) | 42.4  (18.7) | 39.2  (16.1) | 9.0  (2.8) | 9.0  (2.7) | 9.0  (2.9) | 6.3  (4.0) | 3.8  (2.1) | 7.7  (4.3) |
| Melanomas | 63.1  (16.6) | 64.3  (15.7) | 62.1  (17.1) | 10.5  (3.1) | 10.6  (3.1) | 10.5  (3.0) | 10.4  (3.9) | 9.8  (3.0) | 10.8  (4.5) |
| Amelanotic Melanomas | 62.0  (17.6) | 64.0  (17.4) | 60.5  (18.1) | 11.3  (3.1) | 11.3  (2.5) | 11.1  (3.7) | 10.4  (3.1) | 11.4  (2.9) | 8.0  (2.8) |

*M – Males; F – Females
